# Accounting for youth audiences’ resistances to HIV and AIDS messages in the television drama *Tsha Tsha* in South Africa

**DOI:** 10.1080/17290376.2018.1444506

**Published:** 2018-02-06

**Authors:** Blessing Makwambeni, Abiodun Salawu

**Affiliations:** ^a^ Media Department, Cape Peninsula University of Technology, Cape Town, South Africa; ^b^ Department of Communication, North-West University, Mafikeng, South Africa

**Keywords:** audience reception, cultural studies, entertainment-education, HIV and AIDS

## Abstract

Theoretical debates and literature on E-E efforts in Africa have largely focussed on understanding how and why interventions on HIV and AIDS are effective in influencing behaviour change among target communities. Very few studies have sought to investigate and understand why a substantial number of targeted audiences resist the preferred readings that are encoded into E-E interventions on HIV and AIDS. Using cultural studies as its conceptual framework and reception analysis as its methodology, this study investigated and accounted for the oppositional readings that subaltern black South African youths negotiate from *Tsha Tsha*, an E-E television drama on HIV and AIDS in South Africa. Results from the study show that HIV and AIDS messages in *Tsha Tsha* face substantial resistances from situated youth viewers whose social contexts of consumption, shared identities, quotidian experiences and subjectivities, provide critical lines along which the E-E text is often resisted and inflected. These findings do not only hold several implications for E-E practice and research, they further reflect the utility of articulating cultural studies and reception analysis into a more nuanced theoretical and methodological framework for evaluating the ‘impact’ of E-E interventions on HIV and AIDS.

## Introduction

The E-E strategy, conceptualised as the strategic placement of educational content in entertainment education, has gained currency in contemporary health communication praxis and scholarship (Govender, 2013; Obregon & Tufte, 2013). Over the last four decades, E-E has been increasingly used to address health and development issues on the African continent (Govender, 2013; Mahoney, 2013). The growing use and appeal of E-E in health communication have been attributed to contemporary global trends towards ‘entertainmentisation’ which have enabled health educators to add lustre to the relatively ‘dour’ fields of health promotion, education, and development (Singhal & Rogers, 2002).

The success of E-E campaigns as a vehicle for addressing development issues has led to the growth in the theoretical treatment and evaluation of the strategy in academic literature (Singhal, 2013). However, an appraisal of E-E literature shows that most studies on E-E efforts in Africa have largely focused on understanding how and why interventions, particularly on HIV and AIDS, have been effective in influencing behaviour change among target communities (see Cardey, Garforth, Govender, & Myklebust-Dyll, 2013; Moyer-Gusé, 2008). Very few studies have sought to investigate and understand why a substantial number of targeted audiences resist the preferred readings that are encoded into E-E interventions. In light of this context, scholars such as Singhal and Rogers (2002) and Dutta (2006) have argued for the need by E-E and health communication researchers to pay attention to the substantial resistances that E-E interventions face in subaltern discursive spaces.

Critical scholars have further argued that although E-E effects research acknowledges the significant resistances that E-E interventions face in subaltern discursive spaces, its positivist orientation renders it incapable of providing a nuanced methodological and conceptual framework for investigating and accounting for such resistances (Singhal & Rogers, 2002; Waisbord, 2015). E-E audience effects research has also been criticised for failing to factor in context, audience lived experiences and identities in the evaluation process (Nyamnjoh, 2006; Obregon & Tufte, 2013). Criticisms of E-E audience effects research has invited calls for a paradigm shift in communication and E-E research, from an exclusively positivist approach, to a nuanced methodological approach that embraces qualitative audience reception research (Dutta, 2006; Obregón, 2005; Waisbord, 2015).

In light of this context, the objective of this paper is to make use of a cultural studies informed audience reception analysis to investigate and account for the oppositional readings that subaltern black South African youths viewers negotiate from *Tsha Tsha*, a popular E-E television drama serial in South Africa that engages young people on issues surrounding HIV and AIDS. This study is an offshoot of a broader study that investigated the production and reception of *Tsha Tsha* by subaltern black South African youth viewers.

### Conceptualising audience resistances

Studies on audience resistances focus on how and why audiences resist media messages. Research on audience resistances can be traced back to early studies (see Blumler & Katz, 1974) in media and communication research that critically noted that audiences are not passive consumers of media content. Such studies began to acknowledge that media texts are in fact cultural battlefields between the media text and situated viewers who critically interpret, select and evaluate the producers’ end product, always in the context of their everyday lives. In this light, research on audience resistances seeks to understand how and why audiences negotiate oppositional readings from media texts.

Unlike the traditional approach to audience research which conceptualises audience resistances as an individual and autonomous process structured by socio and psychological factors, this paper adopts an alternative but nuanced conceptualisation of resistances that is informed by the cultural studies approach to the study of the media. Cultural studies conceptualises resistances as audiences’ oppositional readings of media texts which result from their complex interaction with the media text (see De Block, 2012). The strength of this approach lies in its ability to link audiences’ oppositional readings of media texts to their identities, ideological, social and cultural contexts of consumption. Instead of viewing media consumption as an autonomous and automatic function as postulated by info-processing theories of socio-cognitive psychology, the cultural studies approach is built on an understanding of audiences as plural in their decoding and reading as a variable process (Livingstone, 2007, 2015).

## Review of the literature

Audience research plays a key role in E-E campaigns in evaluating programme impact on target audiences. Most studies in the mainstream E-E literature have sought to understand how and why E-E interventions have been effective. Slater and Rouner (2002) and Moyer-Gusé (2008) note that minimal attention has been directed towards understanding the resistances that E-E messages encounter in subaltern audiences discursive spaces. The focus of research on understanding how and why effects occur is argued to have led to some scholars to even speculate that E-E interventions carry unitary meanings that are ‘unproblemmatically’ transmitted to the audience (see Moyer-Gusé, 2008).

Although E-E research acknowledges that interventions face some resistances at the message reception end (Singhal & Rogers, 1999, 2002), these audience resistances are considered to be extremely rare (Singhal & Rogers, 1999). Audience resistances have been observed in a wide range of E-E interventions like the popular comedy, *All in the Family* and the Indian television soap opera *Hum Log* (Singhal & Rogers, 1999). Despite the growing acknowledgement of the substantial resistances that E-E messages encounter in audiences discursive spaces, these resistances have not received much attention from theoretically based E-E research.

Although several studies attest to the effectiveness of E-E as a communication strategy used in development efforts (see Govender, 2013; Obregon & Tufte, 2013; Singhal, 2013) of late, a growing number of scholars have begun to question the effects of E-E and development communication interventions as well as the methodologies used to evaluate them (see Dutta, 2006; Mahoney, 2013; Obregón, 2005). E-E evaluations have been criticised for a variety of reasons. First, E-E studies have primarily focused on effects and effectiveness, and power at a time when critical audience research has produced little convincing evidence to suggest media potency and audience passivity.

Second, E-E effects studies, like mainstream development communication research, have not attempted to relate media content to audience behaviour and attitude change beyond the simplistic modernisation; powerful effects approach (see Dhoest, 2015; Nyamnjoh, 2006). Third, E-E effects research has not been able to account for audience resistances in the different contexts in which these resistances occur. This is in spite of the fact that E-E is only one of many competing, and conflicting discourses that may exist in a given discursive space (Singhal & Rogers, 2002).

Fourth, mainstream E-E effects research has been largely hamstrung by its conceptualisation of audiences and human behaviour which has largely reposed on predominant models in health communication such as the health belief model, and the theory of reasoned action (Krumeich, Weijts, Reddy, & Meijer-Weitz, 2001). These cognitive models which are rooted in social psychology and health psychology assume that an individual’s perceptions about the susceptibility and severity of a health threat (perceived advantages and disadvantages of preventive actions and perceived barriers) determine their willingness to change. In recent years, these assumptions have been criticised for their strong emphasis on individual cognitive processes and limited focus on the embeddedness of human behaviour in cultural contexts and social structures (Dhoest, 2015; Krumeich et al., 2001). In this light, E-E effects theorisation locates audience resistance at the individual level, based on the assumption that individuals make autonomous choices outside of structural, political and economic limitations. Audience reception studies have debunked this assumption and shown that audiences do not approach media texts as singular individual members. Rather, audiences are also plural in their readings of media texts. They consist of diverse groups that may have different and at times conflicting views.

Fifth, at the methodological level, E-E effects and health communication research has been criticised by critical scholars for maintaining a predominantly positivist epistemology with audience surveys, experiments and quasi-experiments dominating most evaluation research (Dutta, 2006; Obregón, 2005; Singhal & Rogers, 2002). The exclusive use of quantitative methods in E-E effects studies has resulted in the elision of context and audiences’ personal responses and inner feelings in attempts to theorise programme ‘impact’. The neglect of context and identities in theorising effects has resulted in the ‘superficial’ presentation of findings that are often narrowly generalised to the total population and regarded as indicators of programme impact. Consequently, audience effects studies tend to generalise audience effects to some theoretical population rather than understanding them in terms of the specific context of consumption (see Barbie & Mouton, 2001; Livingstone, 2015).

The shortcomings of positivist E-E effects research has prompted scholars such as Obregón (2005), Nyamnjoh (2006) and Mahoney (2013) to call for a paradigm shift in the evaluation of E-E effects. Critical scholars have advocated for the articulation of cultural studies and reception theory in the evaluation of E-E effects. Cultural studies and reception theory overcome the limitations of empirical E-E effects studies by viewing audiences as social and cultural subjects whose behaviour and attitudes are mediated by the complex reality of everyday life (Silverstone, 1990). This emergent critical approach seeks to overcome the limitations of mainstream E-E effects research which conceptualises audiences as passive and the E-E text as powerful. Instead, the emerging critical approach acknowledges the agency of subaltern audiences and their active negotiation of meaning within their complex and oppressive environments (Dutta, 2006; Livingstone, 2015).

Although a number of studies in health communication (Dutta, 2006; Mahoney, 2013) have been calling for an approach that studies E-E effects through the lens of cultural studies and reception analysis, the study of media effects using reception analysis is not necessarily new in media studies and health communication. Research in mainstream media studies shows a growing emphasis on reception studies over the past 40 years (Obregon & Tufte, 2013; Obregón, 2005). The notion of active audience in cultural studies and audience reception studies has led to a widespread rejection of the exclusive use of quantitative studies (see Hall, 1980). This has seen research on Latin American soap operas being largely undertaken from an audience reception perspective. Unlike mainstream E-E effects research which overlooks context, identities and human agency in the consumption of E-E interventions, Latin American researchers place greater value in audiences’ capacity to negotiate and resist ‘preferred readings’ encoded into health communication texts (Obregón, 2005).

### The turn to cultural studies in E-E effects research

The cultural studies approach the study of the media provides a peculiar and more nuanced conceptual and methodological approach for exploring as well as accounting for youth viewers resistances to E-E messages. The approach is transcendental in so far as it builds on the limitations of early ‘audience effects’ theories to extend its investigation of audience consumption of media texts. Its investigation of the consumption moment in the cycle of culture includes an examination of the lived circumstances, sub-cultural and socio-economic differences that invariably shape the ways in which audiences interpret their experiences of media texts using shared cultural codes (Dhoest, 2015; Morley, 1989).

Unlike the dominant paradigm in E-E effects research which emphasises textual power, the cultural studies approach to the study of the media seeks to examine this power. The approach posits that the belief that the media can affect audiences in some direct or measurable ways has passed (Livingstone, 2015; Silverstone, 1990). Of note, the cultural studies approach views media texts as polysemic and indeterminate. It argues that meaning should be conceptualised as a site of contestation and struggle. The acknowledgement of the indeterminacy of the media text, by cultural studies, opens up the possibility of oppositional readings in the consumption of media texts. By foregrounding the interpretative freedom of audiences and insisting that audiences are not duped but should be viewed as active decoders who will not necessarily accept the positions offered by the text, cultural studies offers an interesting premise for examining the resistances that E-E interventions encounter in subaltern spaces (see Strelitz, 2000).

In this light, cultural studies advance a unique approach to the study of E-E effects. While E-E effects studies approach media texts and audiences in isolation, the cultural studies approach decentres the text by examining the socio-historical and political context in which it is consumed. The context in this instance includes the cultural features, the contexts of immediate situations, domestic context as well as the larger historical context of consumption. Cultural studies, therefore, views the context as well as the textual form, rather than the individual, as vital in understanding the meanings and transformations that the text encounters in the reading process. This way, the media is viewed as constantly mediating culture, as well as being mediated by culture as lived experience (Tomilson, 1991, p. 61).

Notably, the cultural studies approach insists that resistances to media texts can only be understood in the context of the entire social formation (Dhoest, 2015; Williams, 1965). This shift in the study of media ‘effects’ dictates that any attempts to understand audience resistances should begin by situating the audience in their contexts, as it is in these contexts that meanings (and resistances) are made. By foregrounding context in the study of ‘media effects’, cultural studies adopt a conceptual approach that enables researchers to relate audience readings to the wider economic and ideological formations in which these practices take place (see Strelitz, 2000, p. 64).

### Appropriating audience reception analysis

This paper contends that while cultural studies provide a nuanced conceptual framework for investigating and accounting for youths oppositional readings of *Tsha Tsha*, audience reception analysis advances a complementary interdisciplinary methodology for examining audience interpretations and resistances. Unlike empirical E-E effects research which overlooks context in its analysis of E-E impact, the strength of audience reception analysis lies in its interest in locating the subject both historically and socially while acknowledging that audiences are capable of negotiating meaning, making sense of messages and re-editing them using their cultural repertoire (Martín-Barbero, 1993). More so, unlike effects studies that focus on either the production moment or the consumption moment in the ‘circuit of culture’, audience reception analysis combines a qualitative approach to media as texts with an empirical interest in the recipients as the co-producers of meaning (see Dhoest, 2015; Jensen, 1988).

Consequently, audience reception analysis’ nuanced and multi-pronged approach to studying cultural products reinvigorates methodological discussions about ‘media effects’ by positing that both contexts of media production and reception set the limits and boundaries of interpretation. These boundaries can be based on class, socio-cultural, socio-historical, and socio-economic practices (Moores, 1993; Morley, 1989). By categorically affirming that situated cultures provide audiences with resources for the negotiation and resistance of the dominant meanings encoded into media texts, reception analysis has become a useful methodology for analysing the ways in which situated audiences resist the encoded meanings from media texts (see Hall, 1980; Radway, 1984).

Following the recommendations of cultural studies and audience reception studies, this paper locates *Tsha Tsha’s* audiences both historically and socially in order to investigate and account for their negotiation of the HIV and AIDS issues in the television drama.

### The television drama: *Tsha Tsha*

The E-E television drama *Tsha Tsha* is a Xhosa language (with English subtitles) multi-part series focusing on young people living in a world affected by HIV and AIDS and other social problems (Parker, Ntlabati, & Hajiyiannis, 2005). It was initially developed to target youth audiences between the ages of 18 and 24 in South Africa but was later expanded to include all young adults. *Tsha Tsha* was developed and produced jointly by the Centre for AIDS Development, Research and Evaluation (CADRE) and Curious Pictures with technical support from the Health Communication Partnership based at the Johns Hopkins Bloomberg School of Public Health/Centre for Communication Programs. The television drama has been supported financially by the President’s Emergency Plan for AIDS Relief through the United States Agency for International Development (USAID). *Tsha Tsha* has been broadcast on SABC since April 2003 (Hajiyiannis & Jugbaran, 2005).

The television series is set in the fictional town of Lubusi, a small rural town in the Eastern Cape and explores young people’s lives as they transition to adulthood. HIV and AIDS is portrayed in the series along a continuum of aspects including prevention, care, support, treatment and rights. The situations portrayed include living in a resource-constrained environment; caring for sick family members; learning one’s HIV status, living with HIV; disclosure of HIV status and safer sexual practices (Parker et al., 2005). Although the intervention is mainly television based, DVDs are also used to entertain and educate young people in schools and prisons about issues surrounding HIV and AIDS in South Africa.

### *Tsha Tsha*’s macro context of consumption

*Tsha Tsha* is produced and negotiated within South Africa, a country with the largest burden of HIV and AIDS in the world (Zegeye, 2008, p. 25). According to Statistics South Africa (2014, p. 3), the number of people living with HIV is estimated at approximately 5.51 million with 16.8% of adults aged 15–49 being HIV positive. However, it is worth noting that although HIV and AIDS afflicts all racial groups in South Africa, marginalised black people living in rural and urban informal settlements are at the highest risk of infection. The race and class character of HIV and AIDS in the country shows that inequality and poverty are the main structural factors determining vulnerability to HIV infection (Metropolitan, 2006, p. 20).

Of the marginalised black South Africans that are exposed to HIV, it is mostly young people who are most vulnerable to infection (Zegeye, 2008). Youths’ vulnerability and continued exposure to HIV and AIDS are attributed to a highly contradictory and complex South African socio-cultural and economic context in which they imagine and re-imagine their identities; define and redefine risky and normative intimacy; as well as sex and love (Motsemme, 2007, pp. 61–67). Vulnerability to HIV is further worsened by the continued racial-based disadvantages such as exposure to poverty, poor education, limited job opportunities, inadequate skills and training as well as lack of access to services (Zegeye & Maxted, 2002, p. 13).

### Masculine and feminine identities in black South African communities

Masculine and feminine identities that constitute *Tsha Tsha’s* black youth viewers play a key role in meaning making as they provide frames of references for negotiating media texts. Connell (2005) posits that such references shape meaning making especially when viewers encounter representations of men and women whose values oppose or reinforce hegemonic ones. Black youths’ masculine and feminine identities in South Africa have been invariably shaped by historical and social processes such as apartheid and the post-1994 democratic dispensation. Consequently, identity formation in South Africa does not reflect a single and clear response to gender conditions but one that reflects fluidity.

According to Morrell (2001) and Morrell, Jewkes, Lindegger, and Hamlall (2013), hegemonic black South African masculinities tend to emphasise violence as a result of emasculation of political power and impoverishment during apartheid. The colonial system dislocated black South African men’s idea of masculinity which was previously exercised through material and social achievement. The dislocation was achieved through confining black African men to employment in menial and seasonal jobs that could not earn them a livelihood. As a result, black men’s masculinities could only be exercised in the domestic space through the subordination and control of black women (Jewkes & Morell, 2010, p. 4). However, the post-1994, democratic dispensation in South Africa has witnessed the emergence of new masculine identities that promote women liberation and oppose hegemonic prescriptions of ‘exemplary’ masculinity. This emergent ‘new man’ is contradictory and mired in a complex situation where the seeking of ‘new ways of being’ is consistently undercut by an urge to cling to the old ways (Morrell, 2001).

On the other hand, black feminine identities appear peculiar in that they have been shaped through a double-pronged fight against both local and imperialist patriarchies (McClintock, 1993). Ideas about successful femininity in black African communities have emerged within a context of deprivation and violence where economic and physical vulnerability and dependence drive women to a life of compliance, obedience and meekness to men (Connell, 1987; Jewkes & Morell, 2010, p. 4). As a result, hegemonic femininities, in Xhosa culture, emphasise women’s subordination to men through the accommodation of their interests and desires. This subordination is reflected through society’s apportionment of status to mothering children and women’s ability to ‘keep’ a permanent partner and extends to a point where women’s exercise of knowledge about sexual issues is condemned (Krumeich et al., 2001, p. 125).

Consequently, successful femininity in black South African communities in South Africa is largely proven through being desirable to men (Jewkes & Morrell, 2012; Motsemme, 2007). It encourages risky practices such as being wooed with gifts by men; exchanging money or other services for sexual favours; flirting and meeting with boyfriends as avenues for circumventing boredom and poverty as well as acquiring social status and self-esteem in this resource-starved setting (Dunkle et al., 2007). Jewkes and Morell (2010, p. 6) have also observed that one of the entrenched social practices associated with both hegemonic masculinities and femininities in Xhosa culture is that of having *Kwaphenis* (concurrent sexual partners even in marriage). It is a practice located at the centre of HIV transmission in marginal black South African communities.

However, it is also significant to note that the emergence of the ‘new man’ in black communities in the post-apartheid era has also been matched by emergent ‘new femininities’, that exercise independence and manifest tendencies leaning towards ‘explicit eroticism’ (Barlow et al., 2005). Although this ‘new woman’ depicts gender change and feminine agency, what remains unknown is whether she seeks economic and political emancipation rather than just sexual independence (Jewkes & Morell, 2010, p. 5).

### The immediate context of *Tsha Tsha*’s consumption

The sample of youth viewers who participated in this study was purposively and conveniently drawn from the Alice campus of the University of Fort Hare (UFH) in the Eastern Cape province of South Africa. UFH was selected ahead of other locations for several reasons. First, the researcher had easy access to the institution and students having previously studied and taught at the university. This made it possible for the researcher to draw participants from naturally existing communities at the institution. Second, UFH as a previously disadvantaged university with limited resources and a complex socio-historical experience of marginality provided a cohort of students whose characteristics (poor, black, Xhosa-speaking youths from inferior, and underperforming ‘apartheid shaped’ schools in the Eastern Cape with either a township and or former homeland background) were consonant with the study’s population of interest. UFH students’ characteristics did not only qualify them as subalterns within a Manichean post-apartheid South Africa but provided potential lines along which they could resist the dominant meanings in *Tsha Tsha*.

The Alice campus of UFH is located in the former independent ‘Bantustan’ of Ciskei, which is considered to be the poorest province in South Africa. The Nkonkobe municipality in which the campus is situated, and draws its students from, is blighted by high levels of unemployment and poverty with approximately 74% of the populace classified as indigent (UFH, 2009–2016, p. 20). UFH mirrors the intricate history and contradictions of a modern South Africa and is yet to recover from the Apartheid government’s policy of separate education. The policy had cataclysmic effects on UFH in so far as it changed the outward-looking, non-racial and continental outlook of the university into a socio-political and economic morass (Massey, 2001). Despite having a legacy of anti-colonialism, UFH remains in a parlous state attributable to the inequitable treatment during the apartheid era, continued racial and economic marginalisation, poor leadership and declining student numbers (UFH, 2009–2016, pp. 15–16). The ongoing atrophy of UFH results from a vicious cycle of deprivation, with the majority of black South African students getting enrolled into the university coming from former DET schools which perpetuate inferior, inward-looking and disempowering education.

## Method

This study used audience reception analysis to investigate and account for black South African youths’ resistances to HIV and AIDS messages in *Tsha Tsha*. The study focused on series 1–3 which were available in DVD format from CADRE, during the research period. The three series (series 1–3) also constitute the sample that CADRE and its partner organisation DramAidE had made available to higher education institutions in South Africa at the time of the study. A three-stage design process was followed. The first stage sought to understand the encoded text. The second stage gleaned audience readings of the encoded text. The stage of the research design compared the encoded text with audience readings. It further accounted for audience resistances by relating oppositional readings to audiences’ subjectivities and the social context of consumption.

The first stage of the study consisted of a qualitative thematic content analysis of the three series and programme documents in order to understand the encoded text as recommended by Morley (1992) and Schroder, Drotner, Kline, and Murray (2003). Qualitative content analysis and document analysis were used to identify the key themes encoded in *Tsha Tsha*. This process served to prepare the researcher for facilitation of focus group discussions and in-depth interviews with youth viewers. The key themes identified through qualitative content analysis and document analysis played a vital role for the critical comparative empirical analysis of ‘media discourses’ with ‘audience discourses’ in the reception analysis.

After identifying key thematic issues and lessons in *Tsha Tsha*, the second stage consisted of an audience reception analysis which was undertaken to compare the ‘media text’ with the ‘audience text’. To understand the audience text, a pared-down version of ethnography which uses the basic techniques of observation, open-ended interviews and group discussions in combination was used (see Deacon, Pickering, Golding, & Murdock, 1999). Youth audiences viewed all the episodes in the three series as they would in normal viewing. They were shown three episodes continuously, followed by focus groups discussions after which they would view another three episodes followed by discussions until all the three series were viewed and discussed. Intervals of three episodes were considered a logical alternative given the need to balance continuity of viewing with possible lapses in concentration.

A total of 12 focus group discussions were conducted with each of the 2 groups that were studied. The number of focus group discussions was determined by saturation points during the group discussions. Although generalisability is not a key issue in qualitative audience research, a total of 38 young adults who were representative of the population of interest participated in the study. Purposive sampling was used to select the participants. Particular focus was placed on symbolic connection through prior exposure to *Tsha Tsha* and shared cultural characteristics. Key criterion used includes age, ethnicity, schools previously attended and home background. The youth viewers were drawn from black Xhosa-speaking students between the ages of 18 and 24 who constitute *Tsha Tsha’s* primary target audience. Recruiting students with a former homeland background or township background further ensured that the respondents qualified as subaltern in South Africa, a characteristic that had the potential to generate resistances. One group was heterogeneous (gender diverse) while the other was homogenous (in terms of gender) in order to cater for sensitive issues during the discussions. Each focus group consisted of between 6 and 10 members in order to encourage greater opportunities for interaction, trust and sharing of experiences as recommended in qualitative research (see Barbie & Mouton, 2001).

In order to prompt and guide the discussions, the moderators made use of a focus group discussion guide which consisted of guiding questions on key thematic issues on HIV and AIDS identified through qualitative content analysis. The guiding questions allowed for expanded discussion of the specific themes and related issues raised in each episode. The key themes and issues discussed include prevention, multiple sexual partnerships, living with HIV, female empowerment, disclosure of one’s HIV status, safe sexual practices and sexual rights among others.

Focus group discussions were followed by semi-structured interviews with selected youth viewers to probe informants on issues that emerged during the focus group discussions (see Macum & Posel, 1998, p. 132). A total of 15 follow-up semi-structured interviews were conducted. Interviews ensured that controversial views, issues and experiences that could not be expressed in a group context could be addressed individually. The interviews were guided by an interview guide. The first part of the guide focussed on the respondents’ personal and social life while the second part focussed on their viewing of the television drama in relation to the controversial, sensitive or salient issues they raised during the focus group discussions. Both the focus group discussions and the individual semi-structured interviews were conducted in a relaxed and spacious office setting. The office arrangements were made to simulate the common room setting where students normally view television. This ensured that the interview setting remained non-bureaucratic.

The third stage consisted of data analysis. The data collected through qualitative content analysis was analysed and coded thematically while the data that were generated through reception analysis were viewed as discursive constructions co-produced by both the researcher and the situated youths. The data gleaned from focussed group discussions and semi-structured interviews were analysed in a descriptive and deductive mode before being coded thematically. Given that the data collected through focus group discussions and in-depth interviews in audience reception studies does not provide finished accounts of the audiences’ experience and interpretations of the media text, the researchers proceeded to analyse the data by way of interpreting audiences’ interpretations of the television text (see Jensen, 1988, p. 4). This analytical phase consisted of relating back the collected data to the social context of consumption, audiences’ subjectivities and the conceptual framework of the study in order to get a more comprehensive and coherent interpretation of audiences’ oppositional readings of the key themes in *Tsha Tsha*.

The findings of the study were validated through the presentation of the findings at two local conferences, and interpretive agreement between the two researchers. Further validation was achieved by allowing youth viewers to cross-check the interpretations in order to judge for themselves accuracy and fairness in reflecting their experiences.

Ethical considerations were prioritised when conducting the study. First, the researchers received research clearance from UFH to pursue the study. Second, all participants who took part in the study were informed about the aims and objectives of the study. Third, participation in the study was voluntary. Participants were informed about their right to withdraw from the study at any point. Fourth, anonymity and confidentiality were an integral part of the study (during and after). As such, pseudonyms were used to protect the identity of the participants. Fifth, participants were briefed about the findings of the study and afforded an opportunity to comment.

## Results and discussion

Results from the reception study indicate that despite its effectiveness in entertaining and educating black South African youths on issues surrounding HIV and AIDS, *Tsha Tsha* encounters substantial resistances among its situated readers. The presence of wide-ranging resistances in the negotiation of *Tsha Tsha* conflicts with empirical E-E effects studies assumption that E-E texts are unproblematically read by target audiences. Rather, what the audience resistances to HIV and AIDS issues in *Tsha Tsha* show is that context is central in audiences’ consumption of E-E texts as ‘it determines the meaning, transformations or salience of a particular subjective form as much as the form itself’ (Johnson, 1986, p. 67). In this light, the meaning of the E-E text cannot be inferred from the position suggested by the text and its producers but rather emerges from the complex interplay between audiences’ social contexts of reception and the formal properties of the text.

### Conceptualising HIV and AIDS

Results from qualitative content analysis and document analysis indicate that *Tsha Tsha* seeks to provide its audiences with a conception of HIV and AIDS which is based on scientific rationality. This discourse seeks to counter the hegemonic discourse around HIV and AIDS in black South African communities which articulates HIV and AIDS to ‘bad luck’ and ‘misfortune’ in ways that perpetuate denialism, invincibility, avoidance of condom use and other unsafe sexual behaviours among youths (see Motsemme, 2007). The preferred discourse in the E-E drama presents the pandemic as a critical but preventable health issue which has had a negative impact on individual families and South African communities. It educates viewers about the causes, prevention mechanisms and impact of HIV and AIDS on individual families and the community through the experiences of principal characters.

Audience reception of *Tsha Tsha’s* lesson on the causes of HIV and AIDS shows significant resistance from youth audiences who negotiate the oppositional reading of the E-E text. The difference between the preferred reading and audience reading is reflected through Luxolo’s negotiation of Andile’s father’s death due to AIDS:

Andile is not supposed to hate his father; the only problem is that he brought the hated disease into the family. When someone dies of AIDS like that we view that person as having been in the wrong place at the wrong time. We feel pity for him, he is just a victim and we could even laugh.

Luxolo’s negotiation of HIV and AIDS is consonant with the hegemonic ‘bad luck’ discourse in subaltern black South African communities which removes human agency from the HIV and AIDS matrix. Instead of attributing Andile’s father’s HIV infection and subsequent death from AIDS to multiple sexual partnerships as ‘preferred’ by the producers, Luxolo views him more as a victim. Luxolo’s resistance of the ‘preferred reading’ inscribed in the E-E text derives from his interpretive community and its shared subjectivities about HIV and AIDS. Being a young man from Mbizana, a rural area in the Eastern Cape, Luxolo’s situated discourse on HIV and AIDS acts as an alternative framework of reference in the meaning-making process (see Hall, 1980, pp. 137–138). In this light, resistance to the preferred reading is indicative of the significant dissonance between the encoders’ preferred reading of HIV and AIDS and his socially constructed discourse on HIV and AIDS (see Newbold, Boyd-Barrett, & Van Den Bulck, 2002: 41) which is constructed and reinforced within a marginal black Xhosa community where frequent exposure to death, poverty and unemployment promotes a discourse of HIV and AIDS premised on invincibility and denialism (Motsemme, 2007, p. 74; Zegeye & Maxted, 2002, p. 13)

### Multiple sexual partnerships

As part of its educational message to young people in South Africa, *Tsha Tsha* links multiple sexual relationships to exposure and vulnerability to HIV and AIDS through the prism of principal characters like Andile and his ‘playboy’ father. By so doing, the E-E drama promotes fidelity in relationships as a key preventive mechanism against HIV and AIDS. However, situated youth viewers’ negotiation of this lesson shows substantial resistance across gender lines. Resistance to the encoded theme of fidelity manifests itself more as admiration rather than contempt for characters who engage in multiple sexual relationships. Youth viewers’ resistance to fidelity in relationships can be accounted for through their socialisation and lived experiences as Xhosa men and women whose culture apportions status to promiscuous men. This regard apportioned to promiscuous men is illustrated through Vuyiseka’s reading:

Andile’s father can be described as *ulewu* (playboy). For you to be viewed as *ulewu* you sleep with as many women as possible. We then view you as a role model, you are a hero and everyone admires you. The person is huge and one way or the other even us girls want to taste this person.

Vuyiseka’s reading of promiscuity does not only denote a clear contradiction between her situated discourse on masculinity and femininity and the preferred discourses of femininity and masculinity encoded in *Tsha Tsha,* it further affirms the cultural studies position that situated discourses provide audiences with resources for negotiating and resisting the dominant meanings encoded into media texts (Mahoney, 2013; Murdock, 1989). In this instance, the discourse preferred by the E-E drama which emphasises one partner is at variance with the hegemonic discourses in the subaltern youth viewers’ marginal context of consumption which normalise flirting and dating several partners (see Jewkes & Morell, 2010, 2012). The youth viewers’ identity as subjects of a culture that views men who are able to ‘conquer’ women as sexually and socially desirable positions them in direct opposition with the dominant meaning encoded in the E-E drama.

As suggested by reception theorists, locating youth viewers of *Tsha Tsha* historically and socially is critical in order to account for resistances to the preferred readings. In this instance, youths’ resistances to fidelity in relationships can be further articulated to two disparate historical moments in South Africa: the rapture of parental authority in the aftermath of the youth revolt against apartheid in 1976 and the entrenchment of a rights-based culture in post-apartheid South Africa (Motsemme, 2007; Zegeye, 2008). The resultant discourse of sexual freedom which emerges provides raw material for resisting the media text as shown through a male viewer Xakiso:

How can Andile have one girlfriend and just marry her, what if he finds out that she does not satisfy him. Just like buying a car, you have to test drive it first, test drive as many cars as you can before you settle for one.

This oppositional reading is shared across gender lines as evidenced through Ngumakhaya, a female viewer:

This thing (vagina) is not meant for one person, I give it to whomever I decide to give it to. You cannot monopolise it.

Evidently, the youth viewers’ resistance to sexual confinement results from the collusion between the customary discourses of sexuality with an emergent rights-based discourse. Jewkes, Morrell, and Christofides (2009) posit that the customary discourse on sexuality in black South African communities conceptualises sexual exploration as a natural activity even during adolescence. The customary discourse resonates with the emergent rights-based discourse on sexuality and femininity which is characterised by the exercise of sexual independence and tendencies leaning towards ‘explicit eroticism’ (Barlow et al., 2005). Youth audiences’ resistance to fidelity in relationships can therefore be accounted for within this social-historical context of consumption where sexuality, womanhood and manhood are constantly being negotiated through the agency of both the customary discourses and modernity in post-apartheid South Africa.

### Secondary abstinence and non-penetrative sex

Audiences’ resistances to the key HIV and AIDS lessons in *Tsha Tsha* underscore the centrality of situated discourses in audiences’ negotiation of E-E texts. The difference between the media discourses and audiences’ situated discourses also comes out prominently through the lesson on secondary abstinence and non-penetrative sex. These lessons are modelled through the principal characters Boniswa and DJ. Black youth viewers’ resistance to secondary abstinence and non-penetrative sex correspond with Morley’s findings in the *Nationwide study* (1986) that showed that audiences’ social positioning and socio-historical context rather than the text itself influence the reading of media texts. In line with their situated discourses, male and female youth viewers of *Tsha Tsha* read secondary abstinence and non-penetrative sex as ‘unfair’ and ‘torturous’ in the context of relationships. This resistance comes out clearly in a female viewer’s response to Boniswa and DJ’s relationship.

What Boniswa is doing to DJ is unfair, making love and reaching that point. What is the point of going that far if she does not want to have sex? Why should someone wait for months to have sex and then after those months the guy is gonna go?

The resistance to secondary abstinence and non-penetrative sex in this instance emerges from youth viewers subjective positioning and lived experiences as subaltern black Xhosa youths whose dominant culture associates sex with actual penetration (see Motsemme, 2007). The rejection of non-penetration and secondary abstinence as viable preventive mechanisms in the age of HIV and AIDS also needs to be related back to audiences’ location within a novel sexual discourse that emphasises sexual freedom and feminine agency in post-apartheid South Africa. Youth viewers’ resistance to lessons on non-penetrative sex and secondary abstinence shows that E-E messages are not always read in the intended manner by ‘passive’ audiences. Rather, these oppositional readings confirm the cultural studies position that audiences actively negotiate meaning from media texts by constantly drawing from their complex and oppressive environments (Dhoest, 2015; Guha & Spivak, 1988). Youth readers negotiation of *Tsha Tsha* further illustrates that the process of meaning making in E-E, epitomises ‘a guerrilla warfare’ where media discourses and discourses of the situated audiences are always contesting for dominance.

### Confronting hegemonic masculinities

As part of its social change agenda, *Tsha Tsha* also seeks to challenge hegemonic masculinities that perpetuate HIV and AIDS in black South African communities. In this regard, *Tsha Tsha* depicts male characters who transcend socially defined roles assigned to them by their community. One of the principal characters through which *Tsha Tsha* challenges hegemonic masculinity is Andile, a caring young man who in spite of his sister’s presence takes over domestic chores at home when his mother is incapacitated by HIV and AIDS. Through Andile, *Tsha Tsha’s* producers sought to change socially constructed gender binaries that have become untenable. Although a sizeable number of male and female youth viewers learned the ‘preferred’ lesson, a number of male and female viewers resisted Andile’s ‘domestication’. This resistance is aptly captured through a male youth viewer, Luxolo, whose situated discourse and lived experience as a conservative Xhosa man from rural Pondoland in the Eastern Cape provides him with raw materials to resist the preferred text:

Andile is behaving in a sissy manner. You cannot be washing your mother’s feet irrespective of her situation. That may not be the best way of seeing things for others but that is not how one should behave in Pondoland.

Youth viewers’ resistance to the alternative masculine identities presented in *Tsha Tsha* is further evidence illustrating that in as much as E-E texts foreground preferred readings, situated discourses and youth viewers’ individual experiences can help them to negotiate oppositional readings. This observation is in line with Hall’s (1980) argument that audiences do not necessarily accept the preferred readings being offered by the media text. Rather, media texts can be read differently by situated audiences whose discourses differ from those encoded into the E-E text. In this case, black youth viewers of *Tsha Tsha* are subjects of a patriarchal Xhosa culture which positions them in direct conflict with the masculine values embodied by Andile.

### Challenging stereotypical feminine ideals

Besides resisting alternative masculine identities ‘preferred’ by *Tsha Tsha*, both male and female black youth viewers of the television drama also contest the ‘exemplary femininities’ presented by the television drama. Qualitative content analysis of *Tsha Tsha* shows that the television drama challenges hegemonic feminine ideals of ‘reservedness’ and ‘meekness’ that perpetuate HIV and AIDS in black South African communities (see Motsemme, 2007). These ideals are confronted through *Sis Wawi*, an assertive woman who speaks openly about HIV and AIDS much to the annoyance of men in her conservative community. Results from the focus group discussions show that audience readings of *Sis Wawi* are largely shaped by subaltern youths’ social context of consumption and hegemonic identities in black Xhosa communities which value obedience and meekness as hallmarks of African femininity (Jewkes & Morell, 2010, p. 4).

*Sis Wawi’s* outspokenness on sexual matters contradicts the subaltern youth viewers subjective positioning within hegemonic Xhosa culture where women are socialised against disclosing any knowledge about sexual issues and to be submissive to their male counterparts. Consequently, youth audiences’ resistance to *Sis Wawi* emanates from the contestation between two opposed discourses of femininity and sexuality. This contestation is reflected through a female youth viewer’s response in a focus group discussion:

This modern world should not erode our culture and norms and values. As a woman even in the bible, the woman is below the husband. He is the head of the house even when you are a manager of a company. On this sex issue Sis Wawi was supposed to refer to the husband for advice. This woman being involved in sex doesn’t bring the right picture in the community. There are norms and values and women should not cross these boundaries.

The female viewer’s resistance to the exemplary feminine discourse modelled through *Sis Wawi* reinforces the cultural studies thesis that the meaning of media texts is not in the text itself but determined socially. Therefore, youth viewers’ resistance to the preferred meaning of the E-E text needs to be viewed more as constructed out of the conjuncture of the text and the socially situated reader (Fiske, 1987; Livingstone, 2015). In this instance, the female reader’s negotiation of the encoded text is influenced by her context of consumption and subjective position as a Xhosa woman whose culture views women as subordinate to men (see Connell, 1987; Stern & Buikemma, 2014). The female reader is a subject of a dominant customary acquiescent femininity which positions her in direct opposition with *Tsha Tsha’s* liberal discourse. It is from this subjective position that she emphatically condemns *Sis Wawi’s* conduct as *amanyala* (disgusting).

### Negotiating sexual rights

Youth audiences reading of the lessons on sexual rights in *Tsha Tsha* further shows that the consumption of E-E texts is a struggle over meaning where the producers attempt to privilege certain meanings while subaltern readers constantly inflect the ‘encoded text’ using raw materials drawn from their context of consumption. This observation is evidenced through audience reading of the theme on sexual rights in *Tsha Tsha* which is fore grounded through Mandisa, a lesbian character who is maligned by the fictional community of Lubusi for her sexual orientation. The dominant text in *Tsha Tsha* positions Mandisa as a victim of a homophobic community that denies her sexual rights. However, audience readings of this theme reflect a clear difference between the liberal discourse of sexual rights encoded into the media text and the audience text which is negotiated within a largely patriarchal Xhosa culture which delegitimises queer relationships (Morrell, 2001). This resistance towards homosexual relationships comes out clearly in a female viewer’s response to Boniswa and Mandisa’s relationship:

If I find out that my friend is a lesbian like Mandisa, I will let her go there and there. I can never accept someone who is lesbian and be friends with them and have no problems with that. The expectation is that one must get married and have kids.

The female reader’s resistance to Mandisa and Boniswa’s relationship is negotiated from her subjective position within a black predominantly Xhosa South African community characterised by a rigidly sustained heterosexual discourse which manifests itself as social apathy towards alternative sexual identities. The resistance to the media text emanates from the dissonance between the ‘preferred’ media discourse on sexual rights and the customary sexual discourse which delegitimises any other forms of sexuality outside heterosexuality. Black youth readers’ resistance to homosexual relationships is shaped by their social context of consumption which refers to gays and lesbians using the derogatory term *imofi* (homosexual). Resistances to the ‘preferred text’ noted in the focus group discussions show that the majority of youth viewers were not interpellated by the liberal rights-based discourse on sexuality which disturbs their situated discourse of sexuality anchored on procreation (see Spargo, 1999, p. 63).

The oppositional readings to gay and lesbian relationships are negotiated within a marginal context in which the only means to survival and status in society is through heterosexual marriage (Motsemme, 2007). In this light, resistance to homosexual relationships needs to be viewed as shaped by a context where being openly gay does not only expose one to violence in the townships but also leads to marginalisation as gay and lesbian tendencies are largely viewed as foreign (Jewkes & Morell, 2010). However, further audience negotiation of the same theme points to the fact that television experience should not be viewed as an isolated event but as part of the subaltern youths’ total experience. This comes out clearly in a male reader’s resistance to homosexual relationships:

I also do not accept gays and lesbians. Society does not approve. It is not just about Boniswa and Mandisa. Even when Senzo kisses Jaison in *Generations* people are disgusted.

By drawing parallels between Mandisa’s predicament and the ‘maligned’ gay couple in South Africa’s leading soap opera *Generations*, Senzo and Jaison, the viewer’s negotiation of the E-E text unravels how intertextuality activates and reinforces resistance to the ‘preferred readings’. The viewer’s resistance to Mandisa and Boniswa’s lesbian relationship is negotiated within the context of consumption through parallel exposure to *Generations*, and other soap operas in South Africa. In this light, resistances to key themes around HIV and AIDS in *Tsha Tsha* invariably show that the social contexts of consumption and other competing media discourses act as intervening variables which insulate audiences from negotiating the ‘preferred readings’. The findings confirm cultural studies central thesis that the meaning-making process is deeply grounded in the specific cultural systems in which audiences are located.

## Conclusion and implications

The findings of the study show that *Tsha Tsha’s* encoded messages were largely opposed to the cultural norms and values of its situated youth viewers. The evidence of substantial resistances in the negotiation of HIV and AIDS messages in *Tsha Tsha* by subaltern black South African youths in the study reinforce findings from previous studies which show that E-E texts like any other media texts, face substantial resistances in subaltern discursive spaces. The findings further show that E-E texts on HIV and AIDS encounter resistances in subaltern locales because the preferred meanings that are encoded into E-E texts often contradict the values, culture, subjective experiences and beliefs of subaltern youth audiences. Consequently, situated youth viewers’ social contexts of consumption, quotidian experiences and shared subjectivities which are consonant with the dominant ideologies in their social context of consumption, provide lines along which the preferred ‘meanings’ are often resisted and inflected.

These findings have several implications for E-E impact, design and delivery:

First, the evidence of youths’ resistances in the negotiation of HIV and AIDS issues in *Tsha Tsha* largely result from the dissonance between the E-E text and the cultural baggage of the situated youths. This amply shows the need by E-E scholars and practitioners to consider the value of embracing culture centred and anthropological approaches as the basis for both E-E interventions and for engaging with audience resistances. Adopting an approach that extends the dominant socio-psychological analytical frame with approaches that articulate cultural and social context of human behaviour in developing E-E interventions will help practitioners to focus less on informing audiences and focus more on improving the cultures and social structures that shape the health behaviours of situated youth readers.

The study’s findings further suggest the need for E-E interventions to make greater use of ethnographic and anthropologically informed formative research in order to design messages and themes that resonate with the lived experiences of situated audiences. A thorough investigation of audiences’ message environment in the design process will expose producers to other conflicting discourses in the environment that could potentially trigger resistances. Tapping into the richness of local cultures and subaltern people’s experiences in the design process is likely to create greater congruence between E-E media discourses and audience discourses resulting in less resistances.

Second, in order to fully understand and account for the substantial resistances that E-E interventions on HIV and AIDS face in subaltern discursive spaces, E-E impact studies need to embrace more nuanced methodological and conceptual approaches like cultural studies and reception analysis which provide a more nuanced theorisation of the complex relationship that exists between E-E texts and audiences. This will strengthen E-E impact studies by supplementing positivist approaches that tend to atomise, generalise and make broad claims about audiences with nuanced approaches that contextualise audiences and view them as people with agency and fluid identities. In this light, meaning making will begin to be viewed as polysemic and approached as a complex, uneven and fluid process rather than a linear one.
